# Review of Rapid Tranquillisation Guidelines Across NHS Trusts in England

**DOI:** 10.1192/bjo.2024.99

**Published:** 2024-08-01

**Authors:** Claudia Chavasse, James O'Neill, Daniel Romeu, Alex Graham

**Affiliations:** 1 University of Leeds, Leeds, United Kingdom; 2 Leeds and York Partnership Foundation Trust, Leeds, United Kingdom

## Abstract

**Aims:**

Rapid tranquillisation – the parenteral administration of a sedating psychotropic – is frequently utilised to manage acute behavioural disturbances. Each mental health trust in England utilises independent guidelines for rapid tranquillisation, which vary geographically in both recommendations for therapeutic agents, as well as the format in which this information presented. Audits have identified that there is currently poor adherence to rapid tranquillisation protocol guidelines; this may be due to a lack of guideline clarity allowing for personal interpretation. This service evaluation aims to determine the clarity and uniformity of protocols outlined in mental health trust guidelines, in addition to analysing the outcomes of guideline testing to identify if there is consistency between policies, or whether outcomes varied depending on the trust guidelines used.

**Methods:**

Five reviewers (of differing positions throughout clinical training) utilised 52 guidelines from each mental health trust in England, as well as Maudsley and NICE. These were assessed using the same fictional scenario, which simulated a common presentation in which the use of rapid tranquillisation is required. Reviewers deduced the most appropriate therapeutic agent according to the guideline, rated the clarity of each guideline and were invited to leave comments highlighting the guideline's useability.

**Results:**

Seven different management plans were generated by the majority of respondents from the 52 guidelines. Lorazepam was the most frequently selected therapeutic agent. Guidelines with better subjective ratings of clarity had more agreement between reviewers, but full agreement between reviewers was only present for 10 out of 52 guidelines. For 11 guidelines, consensual agreement between reviewers was not reached. Qualitative analysis of comments identified the inclusion of past medical history, drug history and flow charts as positive sub-themes. Redundant language, contradictions and the suggestion to seek senior intervention before trialling a second agent were viewed negatively. Many guidelines did not sufficiently emphasise the need for performing an ECG before administering therapeutic agents, such as haloperidol, which may lead to potentially fatal arrhythmias.

**Conclusion:**

There is no national consensus on the most appropriate rapid tranquillisation agents, with the available evidence being interpreted variously by different trusts and organisations. Poor guideline comprehensibility impacts clinician adherence and allows for personal preference to influence choice of drug. Clear guidelines utilising flow charts to succinctly outline relevant doses and absolute contraindications were viewed favourably by reviewers. The findings of this project highlights to relevant stakeholders the attributes that should be implemented when improving guidelines for the future.

